# Effect of utilization of veno-venous bypass vs. cardiopulmonary bypass on complications for high level inferior vena cava tumor thrombectomy and concomitant radical nephrectomy

**DOI:** 10.1590/S1677-5538.IBJU.2014.0371

**Published:** 2015

**Authors:** Ross M. Simon, Timothy Kim, Patrick Espiritu, Tony Kurian, Wade J. Sexton, Julio M. Pow-Sang, Einar Sverrisson, Philippe E. Spiess

**Affiliations:** 1 University of South Florida, Department of Urology, Tampa, FL, USA; 2 Department of Genitourinary Oncology, Moffitt Cancer Center, Tampa, FL, USA

**Keywords:** Venae Cavae, Nephrectomy, Neoplasms

## Abstract

**Purpose:**

To determine if patients with renal cell carcinoma (RCC) with levels III and IV tumor thrombi are receive any reduction in complication rate utilizing veno-venous bypass (VVB) over cardiopulmonary bypass (CPB) for high level (III/IV) inferior vena cava (IVC) tumor thrombectomy and concomitant radical nephrectomy.

**Materials and Methods:**

From May 1990 to August 2011, we reviewed 21 patients that had been treated for RCC with radical nephrectomy and concomitant IVC thrombectomy employing either CPB (n =16) or VVB (n=5). We retrospectively reviewed our study population for complication rates and perioperative characteristics.

**Results:**

Our results are reported using the validated Dindo-Clavien Classification system comparing the VVB and CPB cohorts. No significant difference was noted in minor complication rate (60.0% versus 68.7%, P=1.0), major complication rate (40.0% versus 31.3%, P=1.0), or overall complication rate (60.0% versus 62.5%, P=1.0) comparing VVB versus CPB. We also demonstrated a trend towards decreased time on bypass (P=0.09) in the VVB cohort.

**Conclusion:**

The use of VVB over CPB provides no decrease in minor, major, or overall complication rate. The use of VVB however, can be employed on an individualized basis with final decision on vascular bypass selection left to the discretion of the surgeon based on specifics of the individual case.

## INTRODUCTION

With the increasing use of cross sectional imaging over recent years, the incidence of renal cell carcinoma (RCC) has increased at an average of 2.5% yearly (1). Although this has led to earlier detection of RCC, 5–10% of patients continue to present with tumor thrombi formation in the inferior vena cava (IVC) at the time of diagnosis. The presence of thrombi itself portends a poorer prognosis; however it has been demonstrated that thrombi level I-IV have equivalent 5-year cancer specific survival of 32%-68% after surgical intervention (1–5). Even though surgery is warranted in most patients regardless of the level of thrombus, an increased perioperative complication rate has been observed proportional to the proximal extent of the tumor thrombus. This increase in complication rate is in part caused by the frequent need for vascular bypass to successfully resect bulky level III and IV IVC tumor thrombi (1–3, 5–9).

In an effort to reduce perioperative complications, liver transplantation techniques that do not utilize vascular bypass have been successfully applied for certain level III thrombi. However, there still exists a population of patients that cannot tolerate the decrease in cardiac return after cross clamping of the IVC, and require vascular bypass for successful resection (3, 10–13). Although it is widely accepted that resection of most level IV thrombi must be accomplished with cardiopulmonary bypass (CPB), there is controversy over which type of bypass should be used for level III thrombi and selected level IV thrombi (1, 6–8, 14, 15). In these patients the use of veno-venous bypass (VVB) has been utilized in effort to reduce the risk of perioperative coagulopathy and neurologic and systemic complications associated with CPB (4, 10–11). Most prior studies examining the use of VVB have been descriptive in nature except for a prior peer reviewed study that demonstrated decreased operative time and bypass time when compared to CPB (16). The aim of the present study was to validate this prior study's results at our own institution while taking an in-depth look at perioperative complications among RCC patients with level III or IV IVC tumor thrombi submitted to such surgery on either VVB or CPB.

## MATERIAL AND METHODS

A retrospective study protocol was approved by our institutional review board prior to identifying patients at our tertiary care referral center from May 1990 to August 2011 with RCC and level III-IV IVC thrombi who underwent a radical nephrectomy and IVC thrombectomy on either VVB or CPB. Prior to their operation, a complete metastatic evaluation was conducted which included history, physical examination, and serological studies that included serum creatinine, complete blood count, calcium assessment, and liver function studies. Patients were also screened with chest x-ray or non-contrast computed tomography (CT) or magnetic resonance imaging (MRI), both with intravenous contrast when no contraindication was present (i.e. allergy to contrast or renal insufficiency). This was performed to assess the presence of metastases as well as differentiate the level of the tumor thrombus and carefully clinically stage patients using the American Join Committee on Cancer (AJCC, 2010) classification (17, 18). Additional tests such as bone scintigraphy were performed at the discretion of the referring urologist based on the patient's clinical presentation. The level of tumor thrombus was determined using the Mayo Classification Scale of IVC tumor thrombi (1, 18, 19).

A retrospective chart review was performed for demographics, estimated blood loss (EBL), transfusion of packed red blood cells (PRBC), bypass pump time, operative time, anesthesia time, length of hospital stay and overall survival. Complications were also retrospectively assigned utilizing the Dindo-Clavien classification system (20). All patients with IVC tumor thrombi who did not undergo vascular bypass (n=103) were excluded from the study.

Our surgical technique for resection of level III and IV IVC tumor thrombi has been previously described in the literature and is individualized based on the clinical characteristics of the patient and at the discretion of the multidisciplinary surgical team comprised of a cardiothoracic, hepatobiliary, and/or vascular surgeon (8). Most patients with Level IV thrombi underwent CPB with the exception of those patients in which the tumor thrombi could be manually migrated caudally. In patients with level III tumor thrombi we rely primarily on VVB when vascular bypass is necessary. However, in instances where level III tumor thrombi cannot be adequately controlled at the level of the suprahepatic IVC we typically utilize CPB. In either case the decision to undergo bypass is determined by our multidisciplinary team based in part on the height of the thrombus, magnitude of IVC involvement, bulk of the tumor thrombi, and the anticipated ability of the patient to tolerate cross-clamping of the IVC. After surgical resection, patients were followed routinely every 3–6 months with history and physical examination, serological testing, and radiographic imaging of the chest (chest x-ray, non-contrast CT) and abdomen (CT or MRI with intravenous contrast provided there were no contraindications).

### Statistical analysis

Estimated blood loss, intra-operative PRBC transfusions, post-operative PRBC transfusions, time on bypass, operative time, anesthesia time, length of hospital stay, overall survival (OS), disease specific survival (DSS) and complication rates were compared between the CPB and VVB groups. Comparisons between groups were made using Mann-Whitney U test for continuous variables and Fisher's exact test for categorical variables. The Kaplan-Meier method was used to estimate overall and disease-specific survival from the time of surgery, with comparisons made using the log-rank test. Two patients that died in the peri-operative period were omitted from the survival analysis as they died prematurely on the study. As such an intention to treat analysis was not performed. All p-values reported are two-tailed with statistical significance set when p<0.05. Statistical analyses were conducted using SPSS 21 (IBM Software division, Somers, NY, USA).

## RESULTS

Our patient population consisted of 21 patients that had been treated by nephrectomy and concomitant IVC thrombectomy for RCC utilizing either CPB (n=16) or VVB (n=5). Of this group, 17 patients were classified as having level IV thrombi (81%) and 4 were classified as having level III thrombi (19%). The median age of the population was 64 years (43–84). Patients undergoing surgical resection had an overall good performance status, with 20 of 21(95.0%) patients having an Eastern Cooperative Oncology Group Performance Status (ECOG PS) of 0 or 1. The clinical and pathological characteristics of the two patient subsets (VVB and CPB) are summarized in Table-1.

**Table 1:** Patient Clinical and Pathological Characteristics.

Feature		VVB (n=5)	CPB (n=16)
**Tumor Thrombus Level**			
	Level III	3(60.0)	1(6.25)
	Level IV	2(40.0)	15(93.75)
**Age at Surgery**			
	Median (Range)	45(43–83)	65(53–84)
**Gender M/F**		43/2	8/8
**Extent of Disease at Time of Surgery**			
	N+	2(40.0)	5(31.25)
	M+	0	5(31.25)
**Histologic Subtype**			
	Clear Cell	4(80.0)	7(43.75)
	Papillary	0	7(43.75)
	Chromophobe	0	0
	Not Specified	1(20.0)	3(18.75)
**Nuclear Grade**			
	1	0	0
	2	1(20.0)	2(12.50)
	3	3(60.0)	4(25.00)
	4	1(20.0)	5(31.25)
	Not Otherwise Specified	0	5(31.25)
**ECOG**			
	0	3(60.0)	9(56.25)
	1	2(40.0)	6(37.50)
	2	0	1(6.25)
**BMI**			
	Median (Range)	29.3 (20.9–35.9)	27.6 (19.5–42.3)

**CPB =** Cardiopulmonary Bypass; **VVB =** Veno-Venous Bypass; **BMI =**Body Mass Index, RCC = Renal Cell Carcinoma; **M =** male; **F =** Female; **ECOG =** Eastern Cooperative Oncology Group Status

(Data in parenthesis are percentages)

The type of bypass utilized was not predictive of overall, minor, or major complication rate. These complication rates were determined using the validated Dindo-Clavien classification system. The overall complication rate was 60.0% in the VVB group versus 62.5% in the CPB group (P=1.0, Table-2). Additionally the minor complication rate (Clavien I and II) was 60.0% versus 68.7% (P=1.0) and the major complication rate (Clavien IIIa-V) was 40.0% versus 31.3% (P=1.0) in the VVB versus the CPB group. Notably, two perioperative mortalities occurred in the CPB group (on postoperative days 2 and 13). The death at postoperative 2 day occurred from renal insufficiency and haemothorax formation. The death at postoperative day 13 occurred from sepsis caused by an enterococcus infection. Additionally, the need for post-operative blood transfusions occurred in 0 of the VVB group and 43.8% of the CPB group (P=0.12).

**Table 2:** Overall Complication Rate By Clavien Classification.

Complication By Clavien Classification	VVB(n=5)	CPB(n=16)
Atrial Fibrillation II	0	3(18.75)
Cephalic Vein Thrombus II	0	1(6.25)
Chylous Fistula II	0	1(6.25)
Deep Vein Thrombosis II	1(20.0)	0
Volume Overload II	0	1(6.25)
Pneumothorax IIIa	0	1(6.25)
Cardiac Tamponade IIIb	0	1(6.25)
Myocardial Infarction IV	1(20.0)	0
Pulmonary Embolus IV	1(20.0)	0
Mortality V	0	2(12.5)

Data in parenthesis are percentages. (P=1.0)

Overall, we did not discover any statistical difference in the perioperative characteristics between VVB versus CPB when analyzing median EBL (2300 mL versus 3250 mL, P=0.35), intraoperative pRBC's transfused (6 units versus 8 units, P=0.66), operative time (362 minutes versus 403 minutes, P=0.28), anesthesia time (407 minutes vs. 473 minutes, P=0.18), and length of hospital stay (8 days versus 11 days, P=0.21). There was a trend however, towards decreased total time on vascular bypass in patients undergoing VVB (29 minutes versus 60 minutes, P=0.09). These results are shown in Table-3.

**Table 3:** Perioperative characteristics.

Features	VVB(n=5)	CPB(n=16)	P Value
Estimated Blood Loss (mL)	2300(1300–5200)	3250(900–9000)	0.35
Intra-operative pRBC's (units)	6(4–12)	8(1–38)	0.66
Bypass Time (min)	34 (20–50)	64 (16–138)	0.09
Operative Time (min)	362 (288–478)	403 (248–865)	0.28
Anesthesia Time (min)	407 (300–541)	473 (384–955)	0.18
Length of Hospital Stay (min)	8 (5–10)	11 (2–20)	0.21

pRBC-packed red blood cells

Data is reported as medians with range demonstrated in parentheses.

The median post-operative follow-up for the entire population was 11.93 months (IQR: 5.59–29.92 months). The median OS for the entire population was relatively low at 16.1 months (IQR: 6.3–32.5 months) with a comparable median estimated DSS for the entire population of 20.6 months (IQR: 6.3–84.8 months). Utilization of one form of bypass over the other did not predict OS or DSS. Median OS in the VVB group was 20.6 months versus 10.16 months (IQR: 5.6–84.8) in the CPB group (P=0.80) with 2-year OS rates of 50% (VVB) and 40% (CPB). The overall DSS for the VVB versus the CPB group was 20.6 months (IQR: 6.3–29.9 months) versus 10.2 months (IQR 5.6–84.8 months, P=0.60) with 2-year DSS rates of 50% (VVB) and (50%).

## DISCUSSIONS

In our current study we attempted to determine if any benefit exists in utilizing VVB over CPB in patients undergoing IVC tumor thrombectomy with concomitant radical nephrectomy for RCC. We assessed our surgical experience in conducting high level IVC tumor thrombi (level III and IV) using either VVB or CPB techniques. We have shown that both approaches can be successfully performed safely acknowledging a high peri-operative complication rate in such challenging surgical procedures for locally advanced disease.

**Supplementary Figure 1:**
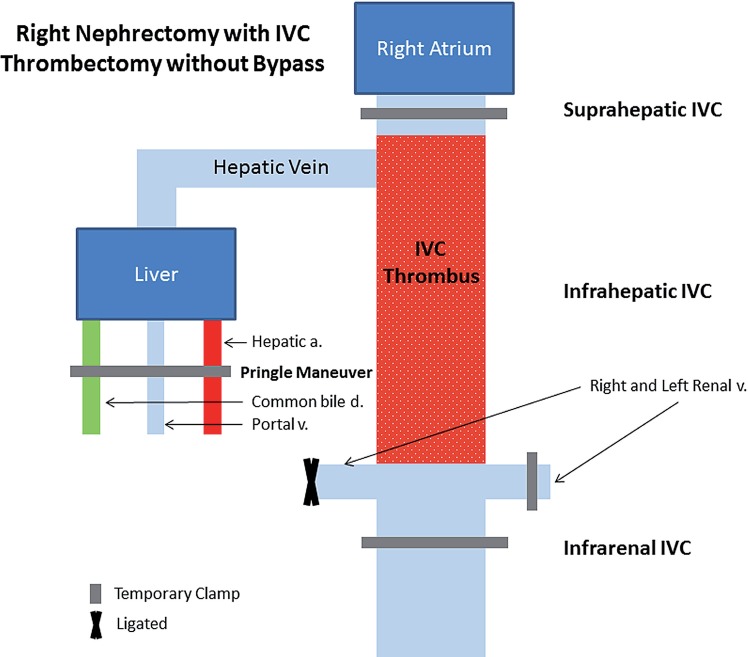
vascular control during right radical nephrectomy with inferior vena cava (IVC) thrombectomy without bypass utilizing the orthotopic liver transplant technique. Temporary clamps are placed on the hepatic hilum (hepatic artery, portal vein, and common bile duct) via the Pringle maneuver, suprahepatic IVC, infrarenal IVC, and left renal vein. If no collateral circulation exists between the suprahepatic IVC and the right atrium, decreased cardiac preload can lead to hypotension.

**supplementary Figure 2:**
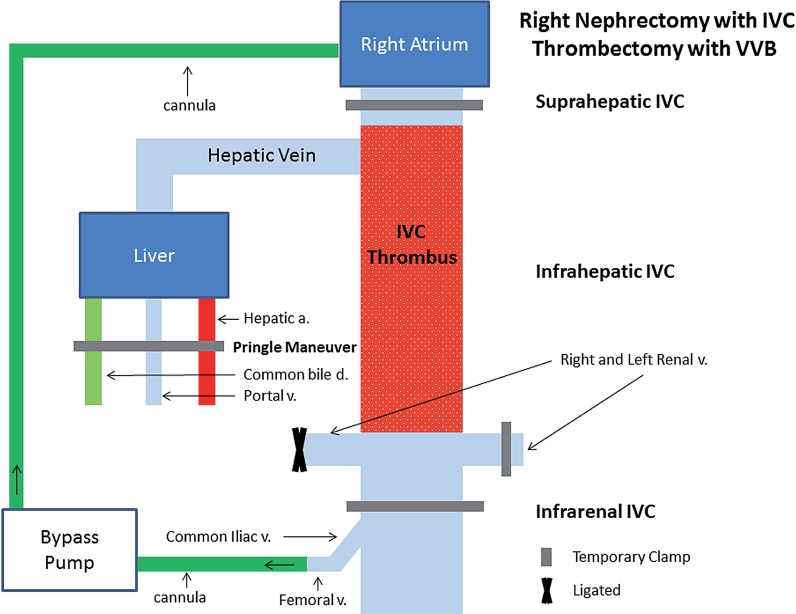
vascular control during right radical nephrectomy with inferior vena cava (IVC) thrombectomy utilizing veno-venous bypass (VVB). similar to the orthotopic liver transplant technique, temporary clamps are placed on the hepatic hilum (hepatic artery, portal vein, and common bile duct) via the Pringle maneuver, suprahepatic IVC, infrarenal IVC, and left renal vein. cardiac preload is restored by the bypass of the portal and venous circulation via cannulation (direction of fow depicted by arrows) of the femoral vein returning blood flow to the right atrium.

Traditionally the use of CPB was utilized in almost all cases of level III and IV tumor thrombi. Due to the known complications of renal and hepatic failure, neurologic dysfunction, postoperative sepsis, and systemic coagulopathy associated with CPB, alternative techniques have been attempted to reduce these complications (1, 16, 21, 22). Some level III thrombi can be successfully managed utilizing orthotopic liver transplant techniques that involves cross clamping of the IVC. This technique was reported by Cianco et al., and reduces the inherent risk associated with vascular bypass. The decrease in cardiac return after IVC cross clamping however is sometimes not tolerable in a select group of patients (Supp. Figure-1). As such, the use of bypass is clearly beneficial and encouraged (Supp. Figure-2). As excessive post-operative bleeding can occur in up to 11% of patients after undergoing CPB however, VVB has been used to possibly reduce the risk of postoperative coagulopathy (24). Initially utilized for liver transplantation, VVB has the advantage that it does not require systemic anti-coagulation, as the cannulas are pre-coated with heparin (16, 22, 23, 25).

The use of VVB in IVC thrombectomy has been described extensively in the literature (10, 11, 16, 26–29). However, only one prior retrospective study conducted by Granberg et al. has compared VVB versus CPB bypass in the setting of RCC and IVC tumor thrombi (16). This study demonstrated patients undergoing VVB (n=13) had significantly shorter bypass, operative, and anesthesia times than did patients treated with CPB (n=28). The study also demonstrated trends towards decreased intraoperative blood loss, reduced transfusion requirements, and a shorter length of hospitalization with VVB. In our current study, we sought to perform an extensive analysis of complications while comparing similar peri-operative characteristics to the previous study. In our study we did not discover trends towards decreased intraoperative blood loss, reduced transfusion requirements, and shorter length of hospital stay with the use of VVB. This could in part be from the limited power of our study or perhaps a selection bias, as any patient undergoing IVC thrombectomy for RCC is subject to substantial blood loss, leading to increased transfusion requirements, and possibly an increased hospital stay. Our study also differed from the previous, (16) as we only demonstrated a trend in decreased time on bypass and showed no statistical difference in operative time and anesthesia time. It could be assumed that since VVB only requires percutaneous access and not direct access to the vasculature like CPB, there would be a decrease in operative and anesthesia time. However since at our institution vascular bypass is reserved for cases that require extensive mobilization and resection of the tumor thrombi, increased operative time, anesthesia time, and time on bypass would be increased in all cases (27).

Prior studies demonstrated comparable survival rates of patients with level III and IV tumor thrombi after surgical resection (5). As evidenced by our current study as well the study conducted by Granberg et al., utilizing VVB versus CPB provides no increase in OS or DSS with one form of bypass versus another. This is conceivable as both modalities allow for adequate resection of tumor thrombi and both involve a substantial and comparable insult to the cardiovascular system. As such, it is quite feasible that if the patient successfully recovers from the perioperative period, there will be no differences in intermediate long-term survival.

We discovered no difference in minor, major, or overall complication rate when comparing VVB versus CPB utilizing the Dindo-Clavien Classification system. Significant major complication rates (Dindo-Clavien IIIa-V) were evident in both the VVB (40.0%) and the CPB (31.3%) group. Notably there were two post-operative deaths (Clavien V) as well as one pneumothorax (Clavien IIIa) and one case of cardiac tamponade (Clavien IIIb) in the CPB cohort. Similarly, one intra-operative myocardial infarction (Clavien IVa) and one post-operative pulmonary embolus (Clavien IVa) occurred in the VVB group. Additionally no difference in minor complications between VVB and CPB were observed. As there is no statistical difference in minor, major, or overall complication rates between the VVB and CPB groups, our study demonstrates that both modalities are associated with significant complications in the perioperative period. Although the use of VVB eliminates the use of systemic anticoagulation, this is only one variable that contributes to post-operative coagulopathy. Consumptive coagulopathy, which is caused by introducing red blood cells to foreign surfaces such as connecter tubing used in both VVB and CPB, increases the expression of tissue factor, which in turn initiates the coagulation cascade leading to consumption of coagulation factors and platelets (23, 24). As substantial post-operative complications can occur when using either VVB or CPB to successfully resect IVC tumor thrombi, knowledge of these complications is paramount for surgical planning and post-operative management.

We acknowledge that our study is limited by its relatively small sample size as well as our single-institution retrospective design. We also acknowledge an inherent selection bias in our study as we utilize a multidisciplinary decision making process and not specific criteria to determine which patients undergo VVB versus CPB. However, in light of this, our study does not necessarily support the use of VVB over CPB in the setting of IVC thrombectomy. As both methods have similar survival and complication rates, the select use of VVB could be employed on high level thrombi (III/IV) on an individualized basis.

## CONCLUSIONS

It has been speculated that the use of VVB could potentially mitigate complications associated with CPB in patients with tumor thrombi undergoing IVC thrombectomy and radical nephrectomy for RCC. However, our study demonstrated that no decrease in complication rate exists with VVB, and that both modalities come with considerable complications that must acknowledged for surgical planning as well as patient education. Although there is no clear-cut benefit to VVB, we discovered a trend of decreased time on bypass, which would possibly be significant in a larger multi-center study. We suggest that CPB is still a valid method for assisting in resection of level III and IV tumor thrombi in patients with RCC, however the use VVB could also be considered on an individualized basis at the discretion of the multi-disciplinary surgical team.
